# Effects of expanded graphite’s structural and elemental characteristics on its oil and heavy metal sorption properties

**DOI:** 10.1038/s41598-024-64695-0

**Published:** 2024-06-14

**Authors:** Divan Coetzee, Thammasak Rojviroon, Sumonman Niamlang, Jiři Militký, Jakub Wiener, Josef Večerník, Jana Melicheríková, Jana Müllerová

**Affiliations:** 1 Department of Materials Engineering, Faculty of Textile Engineering, Technická Univerzita v Liberci, 460 01 Liberec, Czech Republic; 2 Department of Civil Engineering, Faculty of Engineering, Rajamangala University of Technology Thanyaburi (RMUTT), Klong 6, Thanyaburi, Pathum Thani, 12120 Thailand; 3 Department of Materials and Metallurgical Engineering, Faculty of Engineering, Rajamangala University of Technology Thanyaburi (RMUTT), Klong 6, Thanyaburi, Pathum Thani, 12120 Thailand; 4 Department of Materials Engineering (Laboratory Alšovice), Faculty of Textile Engineering, Technická Univerzita v Liberci, 460 01 Liberec, Czech Republic; 5 Institute for Nanomaterials, Advanced Technologies and Innovation, Technical University of Liberec, Bendlova 7, Liberec, 460 01 Czech Republic; 6 Department of Chemistry, Faculty of Science, Humanities and Pedagogy, Technická Univerzita v Liberci, 460 01 Liberec, Czech Republic

**Keywords:** Expanded graphite, Oil sorption, Heavy metal sorption, Crude oil purification, Oil filtration, Materials science, Environmental sciences, Pollution remediation

## Abstract

Expanded graphite has promising potential environmental applications due to its porous structure and oleophilic nature, which allow it to absorb large quantities of oil. The material is produced by intercalating graphite and applying heat to convert the intercalant into gas to cause expansion between the layers in the graphite. Using different intercalants and temperature conditions results in varying properties of expanded graphite. This work has proven that the sorption properties of commercial expanded graphite differ significantly due to the material’s structural and elemental characteristics, which can be attributed to the intercalation method. This resulted in various degrees of exfoliation of the graphite and possible functionalisation of the graphene sheets within the structure. This affected the material's sorption capacity and its affinity for heavy metal sorption by incorporating selectivity towards the sorption of certain metals. It was found that sample EG3, which underwent a less harsh expansion, exhibited lower porosity than EG1, and thus, the sample absorbed less oil at 37.29 g/g compared to the more expanded samples EG1 and EG2 with 55.16 g/g and 48.82 g/g, respectively. However, it was able to entrap a wider variety of metal particles compared to EG1 and EG2, possibly due to its smaller cavities allowing for a capillary effect between the graphene sheets and greater Van der Waals forces. A second possibility is that ionic or coordination complexes could form with certain metals due to the possible functionalisation of the expanded graphite during the intercalation process. This would be in addition to coordination between the metals and expanded graphite carbon atoms. The findings suggest that there is evidence of functionalisation as determined by XRD and elemental analyses. However, further investigation is necessary to confirm this hypothesis. The findings in this work suggest that the first mechanism of sorption was more likely to be related to the degree of expansion of the expanded graphite. Various metals are present in used oil, and their removal can be challenging. Some metals in oil are not considered heavy since they have a relatively low density but can be associated with heavy metals in terms of toxicity.

## Introduction

Expanded graphite has been gaining traction in research due to its unique properties, such as its very high sorption capacity and electrical conductivity, which significantly surpasses its graphite parent material. This is attributed to its highly porous wormlike structure, low density, and easier access to graphene layers, which create conductive pathways for electron flow^1^. This high porosity makes the material ideal for oil spill control since it can absorb large quantities of oil in its pores. Expanded graphite and modified versions of it have been used in wastewater treatment before to act as filters for contaminants such as heavy metal particles; however, there is limited research on the ability of expanded graphite to remove metal contaminants from oil since current methods mainly involve chemical processes such as surfactants or chelating agents^2–4^. The material has many promising novel applications, such as being used as a filler, adsorbent, catalyst, and energy storage^5–8^. The porosity of expanded graphite is greatly influenced by the method of intercalation. The two main methods used to produce expanded graphite are thermal expansion and liquid phase expansion. In the first process, graphite is intercalated using hydrogen peroxide to oxidise the graphite, followed by sulfuric or nitric acid intercalation to form expandable graphite^9^. This is followed by a thermal expansion by heating the expandable graphite to 900–1000 °C to produce the porous expanded graphite. This can be done rapidly or in a programmable manner^10^. The expansion mechanism relies on converting the intercalating agent from the liquid phase to the gaseous phase. This results in an expansion between the graphene layers in the graphite, thus overcoming the van der Waals forces holding the layers together^7,11^. It has been shown that higher expansion temperatures and larger intercalation compounds can be related to greater sorption capacities of expanded graphite due to the formation of larger pores in the more expanded structure^12^. The liquid phase expansion of graphite is less often used because it is more time-consuming than the thermal expansion method. In this method, similar intercalants and oxidisers may be used as in the thermal expansion method, with the possibility of increasing the temperature to promote the expansion. The liquid phase expansion method is less harsh than the thermal expansion method and typically results in more orientated expanded graphite with less damage to the graphene layers in the structure^13–16^. After expansion, some of the intercalator may remain in the expanded graphite structure, most likely in free form between the graphene sheets and the porous structure. Other impurities may be due to the degree of oxidation increasing oxygen species^17^.

**Figure 1:**
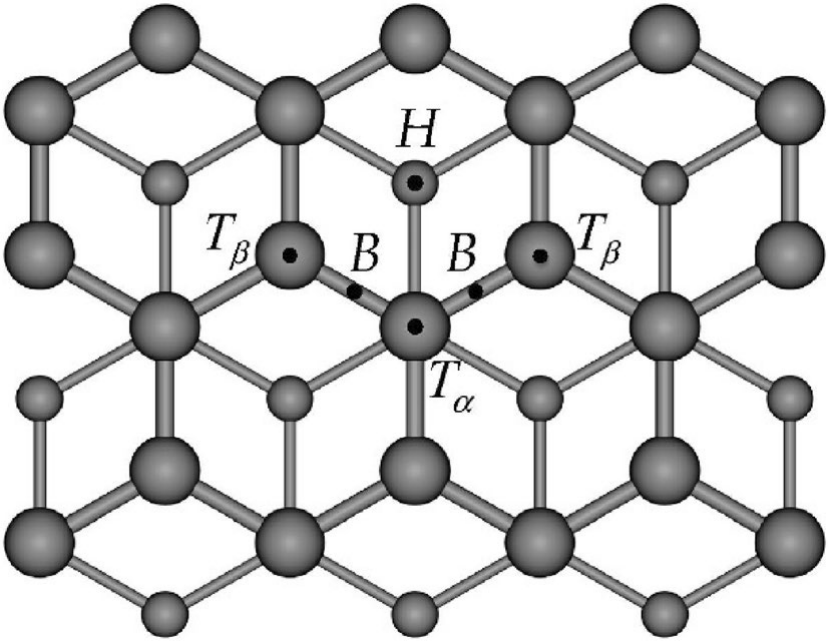
Schematic diagram of the (0 0 0 1) plane, i.e. basal plane, of graphite. The ABA stacking pattern of graphite, also known as the Bernal polymorph, results in two types of surface carbon atoms, one sitting directly above a carbon atom in the layer beneath (α) and the other seated above a void in the layer beneath (β)^29^.

Over the past century, we have heavily relied on oil to sustain our modern lifestyles. In recent decades, we have started to understand more about the environmental impact of this dependence on our ecosystems and the damage it is causing. Except for a few key oil spill disasters that mark our history, we are still under-informed about frequent smaller spills and contamination due to regular transport activities such as the shipping industry^18,19^. Sorbents are one of the most effective materials to clean up oil spills. These are oleophilic materials which attract oil and repel water. The efficiency of a sorbent depends primarily on its sorption capacity and recovery. Other factors such as density, geometry, and wettability are also important, but they can generally be modified or adapted. Sorbents are one of the cheapest methods of cleaning onshore oil spills. However, many of these materials have varying costs and effectiveness and could have a negative impact^20,21^. Petroleum oil is a biogenic mixture of heavy and light oils and contains heavy metals such as nickel and vanadium. Oil used in machinery may contain more heavy metals due to the wear and tear of metal components^22^. These metals are present in the oil in ionic form, forming Ni(II), V(IV), Fe(II), Cd(II) and Pb(II) inorganic salts and organometallic complexes with ligands such as hexanoic acid and 1-propanethiol. These metal ion salts and complexes complicate the processing of crude oil for recycling and could worsen its negative environmental impact^23,24^. Even though some metals in oil, such as aluminium, are not considered heavy metals due to their low density, they are typically associated with heavy metals in terms of environmental toxicity^25^. In the last decade, combining expanded graphite with magnetic metal particles such as iron has become popular. This facilitates the removal of the particles after sorption, and the slight density increase is said to make it less susceptible to being blown away in windy conditions. The combination of the metal particles to the surface of the expanded graphite could explain the good heavy metal sorption observed in previous experiments. This adsorption mechanism is due to intermolecular and electrostatic forces and coordination bonds between the expanded graphite and the metal particles^26,27^. Experimental data has shown that transition metal atoms with greater d-orbital defects have greater adsorption towards graphite^28^. Other than coordination bonds, transition metal elements have varying degrees of adsorption to different sites, as illustrated in Fig. 1. These sites were experimentally determined to be the tops of the alpha and beta carbons (*T*_*α*_*, T*_*β*_), the bridges (*B*) and the holes (*H*). It was noted that the adsorption energy of transition metals, Cr and Au, increases with the number of graphitic layers when accounting for dispersion forces due to van der Waals forces holding the layers together. Therefore, when measuring the adsorption energy of metals, the value measured using a single plane of graphene is said to set the lower limit of the energy measured for these elements^29^.

Oil is absorbed in the porous structure of the expanded graphite due to hydrophobic interactions between the material and the oil. The sorbed oil can be recycled by increasing the temperature of the sorbed particles to release the oil. Other methods of recycling the oil are by mechanical means, such as pressing out the oil from the particles and solvent extraction^21,30^.

## Experimental

### Materials

Three types of commercial expanded graphite (Sorbetin, Czech Republic), EG1, EG2, and EG3, were used. The expanded graphite was produced using sulfuric and nitric acid intercalated expandable graphite in varying ratios, as identified by elemental analysis. Recovered marine heavy oil (RMUTT, Thailand) was used for the experiment (Kinetic viscosity 88.6 cst at 40 °C).

### Methods

The expanded graphite was characterised using Scanning Electron microscopy (SEM) (Vega3 TESCAN) for visual characterisation and X-ray Diffraction (XRD) to determine the average crystallite size and interlayer spacing. Raman Spectroscopy was used to assess the degree of exfoliation. Elemental analysis (Elementar Vario EL cube) was used to determine the basic elemental composition of the three expanded graphite samples. Each sample of expanded graphite was placed in heavy oil using a polypropylene mesh for 30 min and 24 h, respectively, according to the ASTM F 726-99 standard. This was followed by allowing the expanded graphite sample to drip excess oil for 2 min and weighing each sample to determine the total sportive capacity. The excess heavy oil of each sample was analysed (Agilent 7300DV ICP-OES) for its heavy metal content to determine if the expanded graphite removed any heavy metals from the excess remaining oil under the following analysis conditions. 3 replicates, Pump Speed: 12 rpm, Uptake delay: 12 s, Read Time 5 s, RF Power 1.2 kW, Stabilisation Time 15 s, Viewing Mode SVDV, View Height 8 mm, Nebuliser Flow 0.70 L/min, Plasma Flow 12.0 L/min, Aux Flow 1.00 L/min, Limit of Detection: 0.1 ppm.. The viscosity of the heavy oil was measured according to the ASTM D445 standard.

## Results and discussion

### Scanning electron microscopic (SEM) analysis

**Figure 2:**
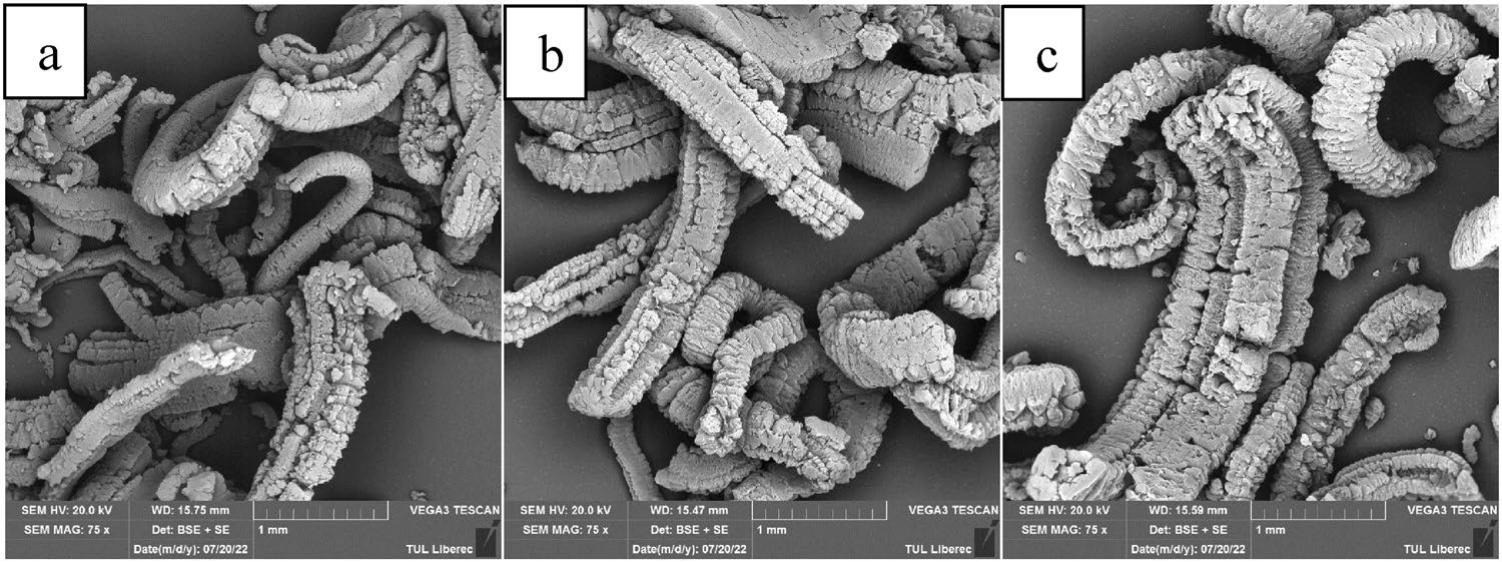
(**a**) SEM images of samples EG1, (**b**) EG2 and (**c**) EG3.

SEM imaging of the three expanded graphite samples used is presented in Fig. 2.

SEM imaging of all three expanded graphite samples showed a significant degree of expansion in the c-axis direction of all samples tested, as indicated by the characteristic wormlike structure.

### Elemental analysis

Elemental analysis shows varying amounts of sulphur and nitrogen in the expanded graphite samples, as illustrated in Table 1.

**Table 1:** Elemental analysis of expanded graphite samples.

Sample	N %	C %	H %	S %	Rest %
EG1	0.96	91.57	0.10	1.41	5.97
EG2	0.80	98.80	0.04	0.30	0.06
EG3	0.11	96.65	0.03	0.60	2.62

It was known that EG1 was primarily produced using sulfuric acid as intercalant, whilst EG2 and EG3 had varying ratios of both sulfuric and nitric acid expandable graphite used before expansion. This would suggest that using sulfuric acid as an intercalant left the highest degree of impurities in the samples, possibly due to the functionalisation of the graphene sheets after oxidation.

### Raman spectroscopy

**Figure 3:**
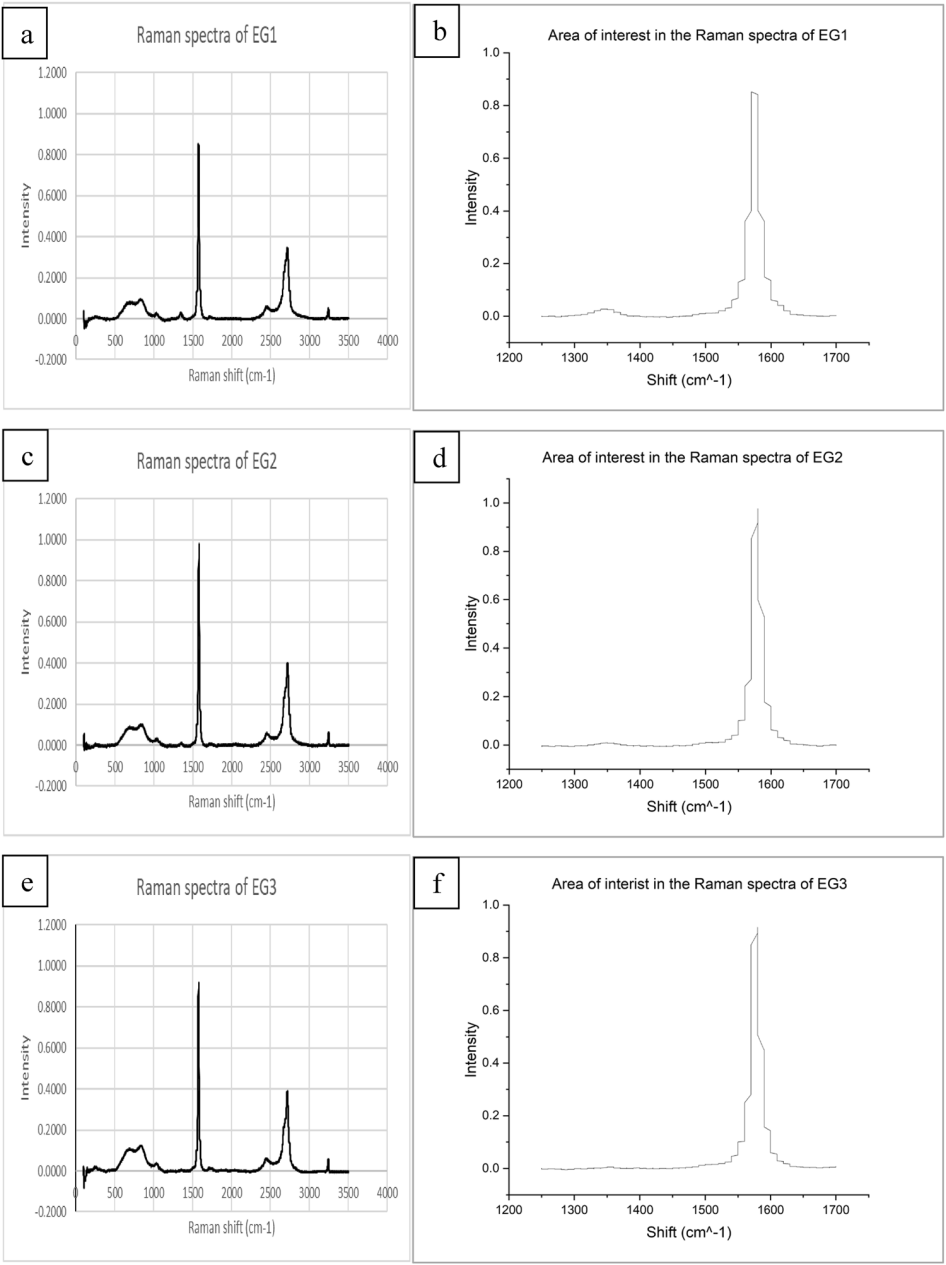
(**a**) Raman diffraction of EG1; (**c**) EG2 and (**e**) EG3 with their respective areas of interest for analysis (**b**–**f**).

Raman spectroscopy for the expanded graphite samples is presented in Fig. 3, with peak integration data in Table 2. The degree of exfoliation of the expanded graphite was determined by the ratio of the peaks of the D-band at ∼ 1350 cm^−1^ and the G-band at ∼ 1580 cm^−1^^31^.

**Table 2:** Raman diffraction peak areas of respective samples.

Peak shift	EG1 peak area	EG2 peak area	EG3 peak area
(P1) 1350–1360	1.46	0.48	0.18
(P2) 1570–1580	21.88	22.63	21.08
Ratio (P1/P2)	0.0669	0.0213	0.0085

It was noted that the peak broadening observed in the aforementioned D1 and D2-bands ∼ 2700 cm^−1^ can be attributed to defects in the graphene planes caused by the expansion during production^28,29^. From the data in Table 2, it was determined that EG1 exhibited the most significant degree of exfoliation with an ID/Ig ratio of 0.0669 compared to 0.0213 and 0.0085 for EG2 and EG3, respectively. This correlated with the sorption capacity of the samples since the higher degrees of exfoliation resulted in a larger oil sorption capacity.

### X-ray diffraction (XRD)

**Figure 4:**
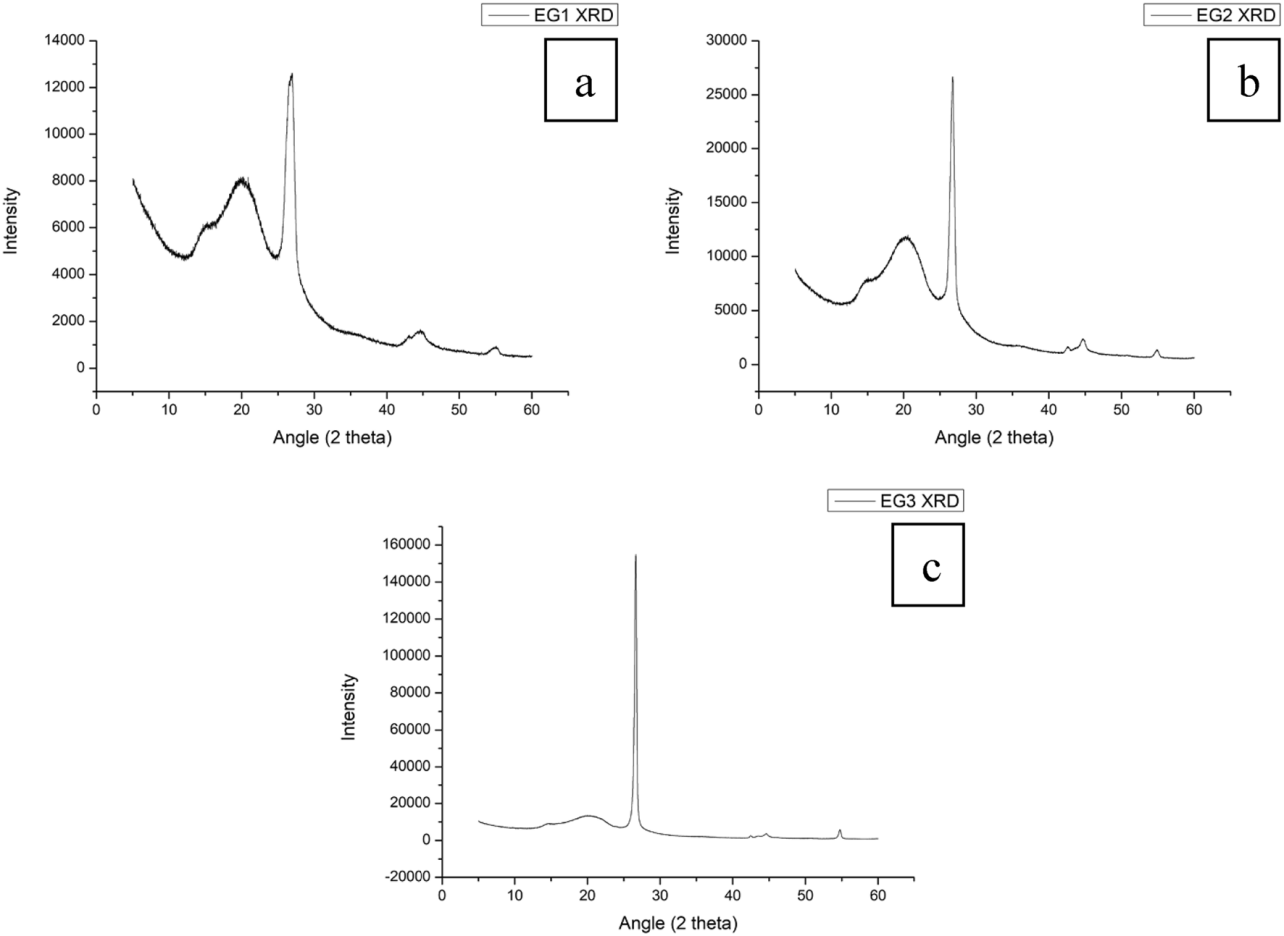
(**a**) XRD of EG1, (**b**) XRD of EG2 and (**c**) XRD of EG3.

XRD was performed on the expanded graphite samples, as illustrated in Fig. 4. The interlayer spacing (d) of the graphite sheets was determined according to Eq. (1), where (n) is the diffraction order, which is equal to 1, (λ) is the wavelength of the incident X-ray source at 1.5406 Å and (θ) the diffraction angle in radians. The average crystallite size was determined according to Eq. (2) where D is the crystallite size in nanometres, K is the Sherrer constant 0.89, (λ) is the wavelength of the x-ray source at 0.15406 nm, (β) is the FWHM (full width at half maximum) in radians and (θ) is the peak angle^31,32^. Data for the interlayer spacing and the crystallite size is presented in Table 3. Since XRD is a structural measurement technique, samples were carefully placed in the sample holder. It was noted from the diffraction pattern that changes in sample weight resulted in an increased signal intensity. However, the peak ratio was unaffected to conclude the measurements.

**Table 3:** XRD peak processing data to determine interlayer distance and crystallite size.

	Peak angle (2θ)	Peak angle (θ)	Peak area	FWHM	Peak area percentage of total (%)	Interlayer distance, d (Å)	Crystallite size, D (nm)
EG1	15.44	7.72	3653.18	3.06	10.58	5.74	0.05
	19.95	9.98	16,247.74	4.60	47.05	4.45	0.03
	26.67	13.34	12,269.53	1.28	35.53	3.34	0.11
	36.14	18.07	57.59	1.32	0.17	2.48	0.11
	42.78	21.39	168.07	0.87	0.49	2.11	0.17
	44.54	22.27	1749.07	2.50	5.06	2.03	0.06
	54.88	27.44	390.47	1.23	1.13	1.67	0.13
Avg						3.12	0.09
EG2	15.46	7.73	7171.64	3.56	13.14	5.73	0.04
	20.19	10.10	28,804.45	4.54	52.78	4.39	0.03
	26.69	13.35	15,491.65	0.71	28.39	3.34	0.20
	36.17	18.08	284.75	2.00	0.52	2.48	0.07
	42.75	21.37	554.96	1.13	1.02	2.11	0.13
	44.65	22.32	1773.92	1.42	3.25	2.03	0.10
	54.85	27.42	493.76	0.70	0.90	1.67	0.22
Avg						3.11	0.11
EG3	14.66	7.33	2790.93	1.73	2.70	6.04	0.08
	19.91	9.96	38,691.69	6.10	37.36	4.46	0.02
	26.61	13.30	56,500.47	0.38	54.56	3.35	0.37
	42.48	21.24	479.36	0.42	0.46	2.13	0.35
	43.40	21.70	674.09	0.73	0.65	2.08	0.20
	44.58	22.29	2338.60	1.15	2.26	2.03	0.13
	54.73	27.37	2082.29	0.42	2.01	1.68	0.36
Avg						3.11	0.22

Bragg's equation^31^:

1 $$d=\frac{n\lambda }{2\text{sin}(\theta )}$$

Sherrer's equation^31^:

2 $$D=\frac{K\lambda }{\beta cos\theta }$$

As seen from the diffraction pattern in Fig. 4, the main crystalline graphite peak in the (002) plane of the expanded graphite was observed ∼ at 26.6°. Integration of this peak showed that EG3 had the highest degree of crystallinity of all three samples at 54.56% compared to 28.39% and 35.53% for EG2 and EG1, respectively. This was also reflected in the average crystallite size, where EG3 exhibited much larger crystal grain boundaries at 0.22 nm compared to 0.11 nm and 0.09 nm for EG2 and EG1, respectively. This would suggest that EG3 maintained most of its graphitic structure compared to EG1 and EG2, which is suggested to have undergone harsher expansion conditions, resulting in reduced crystallite size and increased grain boundaries. The 002 crystalline peaks exhibit an interlayer distance of ∼ 3.35 Å, the same as graphite. In contrast, the broader peaks at lower 2Theta angles show higher degrees of expansion with interlayer distances > 3.34 Å^33^. Slight distortions are present in the crystalline peaks in the diffraction patterns of all three expanded graphite types, as well as additional peaks appearing in the 004 plane, which are typically not associated with graphite. Although distortion in the crystalline peaks could be due to the porous structure causing some distortion in the diffracted X-rays, comparative analysis using the PDF5 + database has shown that the additional diffraction peaks could indicate a different covalent species, which could prove that there is functionalisation of the graphene sheets during the intercalation process with non-carbon atoms. This evidence should be further investigated using techniques such as Solid-State NMR to prove the possible covalent functionalisation of the graphene sheets.

### Total sorptive capacity

**Figure 5:**
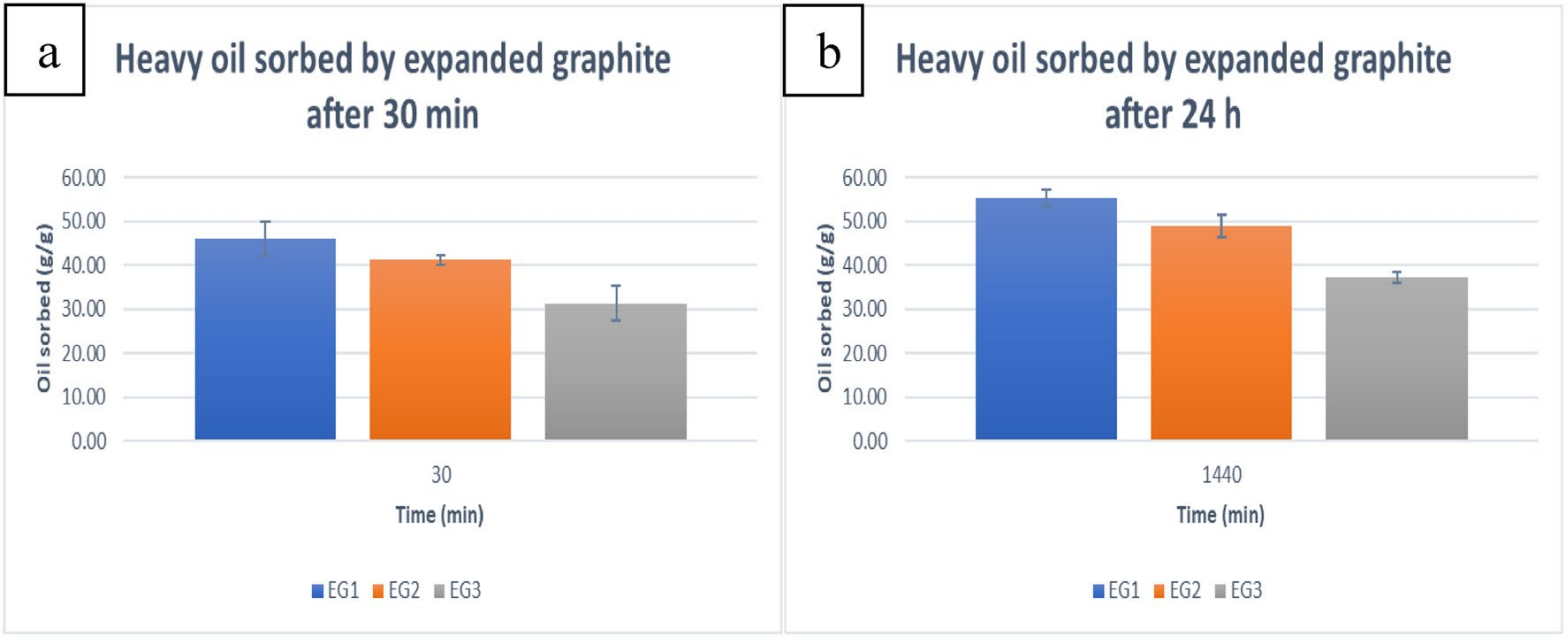
Total sorptive capacity of expanded graphite samples for heavy oil after (**a**) 30 min and (**b**) 24 h.

The total sorptive capacity of the expanded graphite samples is presented in Fig. 5.

From the data presented in the graphs in Fig. 5, it was determined that EG1 exhibited the highest sorptive capacity at 46.12 ± 3.75 g of heavy oil per gram of sample, followed by EG2 and EG3 with 41.16 ± 1.23 g/g and 31.26 ± 4.02 g/g after 30 min, respectively. The amount of oil sorbed after 24 h showed a similar trend in capacity, where EG1 absorbed 55.16 ± 1.90 g/g, which was an average improvement of 19.6%. For EG2 and EG3, the amount of heavy oil sorbed increased to 48.82 ± 2.54 g/g and 37.29 ± 1.30 g/g, showing an 18.6% and 18.7% improvement in average sorptive capacity, respectively. This can be directly related to the degree of exfoliation of the graphite. The higher the degree of exfoliation, the more open pores there are in the structure of the expanded graphite, resulting in a higher sorptive capacity.

### Heavy metal sorption

The metal content of the heavy oil and the content after exposure to the expanded graphite are presented in Table 4.

**Table 4:**
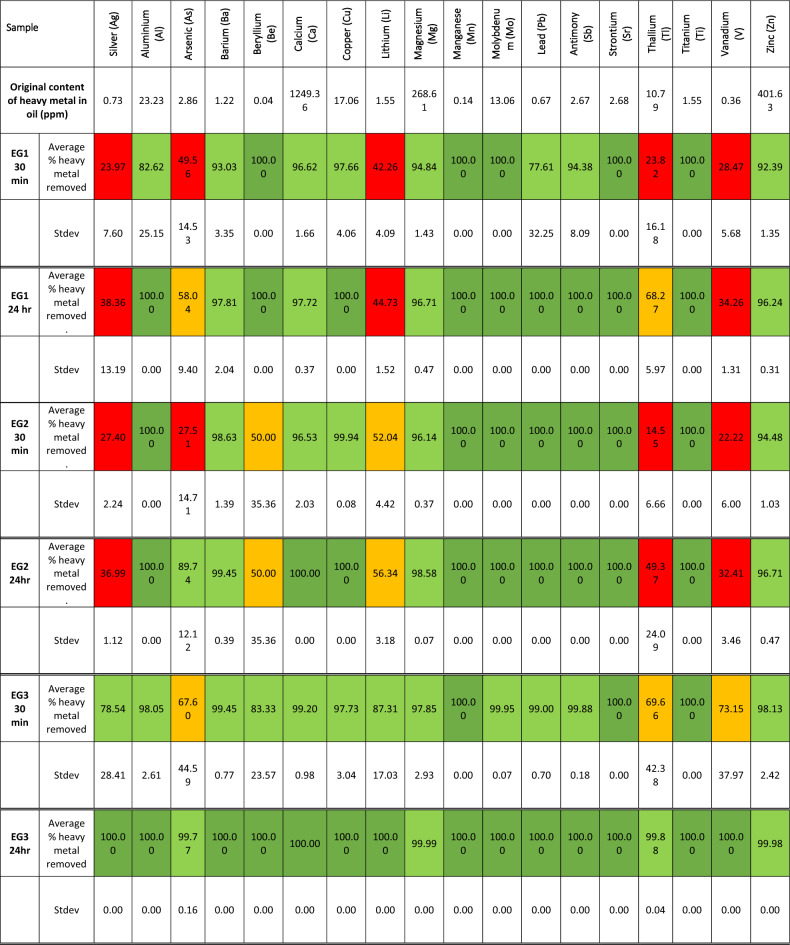
Heavy metal sorption data.

Previous experiments have shown that it was necessary to modify expanded graphite to remove metals from solutions, like decorating the material with metal oxides such as manganese oxide to remove lead and nickel heavy metals^34^. This work has proved that unmodified expanded graphite does have an affinity for lead by absorbing 100% of the heavy metal from excess oil after 24 h for all samples. Even though the average standard deviation for the replicate measurements is 0.00, it should be noted that there is still the possibility of error given the instrument's limit of detection of 0.1 ppm and the amount of metal ions initially present in the heavy oil. EG2 and EG3 performed the best by absorbing 100% and 99.00% of the lead after 30 min of exposure to the oil. EG 1, however, did perform acceptably by removing 77.61% of the lead from the excess oil, but with a significant deviation in results of 32.25%. Overall, EG3 fared the best at removing nearly all heavy metals from the heavy oil. EG2 and EG1 followed this performance since these failed to remove some heavy metals from the heavy oil. EG1 and EG2 could not remove over 60% silver and 65% vanadium. EG1 removed all of the beryllium from the heavy oil compared to EG2, which only managed to remove 50 ± 35.36% and was 18.9% better at removing thallium. EG2 was better at removing arsenic and slightly better at removing lithium after 24 h. There are two possible reasons for this observed difference in heavy metal sorption. The first is the difference in the degree of exfoliation of the expanded graphite samples since it had a noticeable effect on the amount of oil absorbed, which would also provide access for the metal particles to penetrate the expanded graphite structure for entrapment and access to crystalline areas with a higher degree of van der Waals forces. This difference was notable for all three samples, as confirmed by Raman spectroscopy, and suggests a possible trend in the heavy metal sorption selectivity. The second possible reason is the chemical differences due to impurities left after intercalation and the possibility of functionalising the graphene sheets. This difference was most noticeable for EG1 compared to EG2 and EG3 due to the large amount of sulphur present. It is suggested that due to the known sorption mechanism of metals to graphite, the first possible reason is most likely to explain the differences in heavy metal sorption since EG3 has a higher degree of crystallinity compared to EG1 and EG2, meaning that the van der Waals force effect is more significant in the structure of EG3. Some metals prefer this method of sorption to graphite and, therefore, could explain why EG3 could remove certain metals, which EG1 and EG2 could not^26,28,29^. Since these zones of higher crystallinity are spread unevenly throughout the structure of the expanded graphite, areas of higher exfoliation could allow the particles to move towards the more crystalline parts for capturing.

## Conclusion

This work has proven that unmodified expanded graphite can absorb up to 55 times its weight in heavy oil, making it suitable for oil spill control and purification. This ability is accredited to its porous structure provided by its degree of expansion. The XRD measurements mainly look at the unexpanded structure of the expanded graphite, there appeared to be little correlation to its oil sorption capacity except that the lesser expanded structure of EG 3 resulted in a lower sorptive capacity compared to the more expanded and less crystalline EG2 and EG1; however, this does not account for the capillary gap sizes in the expanded part of the material which does influence its sorption capacity. This would explain why EG2 had a lower sorptive capacity compared to EG1, even though it had a slightly smaller crystalline component to its structure. It has been observed in this work that there are differences in the sorption properties of commercial expanded graphite due to the different parameters of intercalation used in its commercial manufacturing process. This does not only apply to the sorption capacity of the material but also its affinity for heavy metals. There are two main reasons for this metal affinity based on the differences observed in this work. Firstly, there is a difference in the degree of exfoliation resulting in a porous structure and the harshness of the method used to affect the degree of exfoliation. This directly influences the sorption capacity and the affinity of some metals to interact with the van der Waals forces in the more crystalline structures since the metal ions can become trapped in the smaller cavities. As hypothesised, a second reason could be the possible functionalisation of the graphene sheets in the graphite structure, resulting in chemical functionalisation, as explained by the elemental analysis, which results in complex formation with the metal ions. Additional peaks appearing in the XRD spectrum for all three types of expanded graphite indicate that there is possible covalent functionalisation of the expanded graphite, which occurs during the intercalation process and is not removed during expansion. It is suggested that the first proposed mechanism has the most significant influence on the sorption properties due to the known mechanisms of heavy metal sorption to graphite. However, the second hypothetical mechanism cannot be ruled out entirely due to the observed selectivity of the sorption properties. The chemical functionalisation of expanded graphite due to intercalation is still relatively unknown; thus, further investigation of its effect is required for future study. This could be performed using techniques such as NMR. However, graphite is notoriously difficult to obtain a proper NMR spectrum from and requires significant background noise suppression. Removal of heavy metals from oil is typically performed using surfactants or chelating agents, which can be challenging to remove from the oil and may result in an unusable byproduct of contaminated surfactant^2,3^. Processes involving these can also be more costly compared to expanded graphite, which costs around 100 Euros per kilogram to purchase and can be reused for the simple filtration of oil without the need for chemical separation.

## Data Availability

All data generated or analysed during this study are included in this published article.

## References

[1] Coetzee, D., Perez Aguilera, J. P., Šubrova, T., Wiener, J., & Militký, J. Expanded graphite to enhance the conductive and mechanical properties of geopolymer, PVA and epoxy. In *Presented at the NANOCON 2023*. 10.37904/nanocon.2023.4780 (2023).

[2] Kholghi N, Amani H, Malekmahmoodi S, Amiri A (2020). Investigation on heavy metal removal from a crude oil contaminated soil using rhamnolipid biosurfactant as a new eco-friendly method. Tenside Surf. Deterg..

[3] Tavakoli O, Goodarzi V, Saeb MR, Mahmoodi NM, Borja R (2017). Competitive removal of heavy metal ions from squid oil under isothermal condition by CR11 chelate ion exchanger. J. Hazard. Mater..

[4] Park J-I (2021). Efficient iron oxide/expanded graphite nanocomposites prepared by underwater plasma discharge for removing heavy metals. J. Mark. Res..

[5] Shi P, Zhu S, Zheng H, Li D, Xu S (2014). Supported Co _3_ O _4_ on expanded graphite as a catalyst for the degradation of Orange II in water using sulfate radicals. Desalin. Water Treat..

[6] Li C, Zhang B, Liu Q (2020). N-eicosane/expanded graphite as composite phase change materials for electro-driven thermal energy storage. J. Energy Stor..

[7] Dong Y, Zhou G, Ding H, Yuan F (2021). Preparation technics and application of expanded graphite. ACE.

[8] Elbidi M, Resul MFMG, Rashid SA, Salleh MAM (2023). Preparation of eco-friendly mesoporous expanded graphite for oil sorption. J. Porous Mater..

[9] He J, Song L, Yang H, Ren X, Xing L (2017). Preparation of sulfur-free exfoliated graphite by a two-step intercalation process and its application for adsorption of oils. J. Chem..

[10] Bannov AG (2021). Highly porous expanded graphite: Thermal shock vs programmable heating. Materials.

[11] Mazela B, Batista A, Grześkowiak W (2020). Expandable graphite as a fire retardant for cellulosic materials—a review. Forests.

[12] Goudarzi R, Hashemi Motlagh G (2019). The effect of graphite intercalated compound particle size and exfoliation temperature on porosity and macromolecular diffusion in expanded graphite. Heliyon.

[13] Jin S (2009). ‘Low-temperature expanded graphite for preparation of graphene sheets by liquid-phase method. J. Phys. Conf. Ser..

[14] Zhu L, Zhao X, Li Y, Yu X, Li C, Zhang Q (2013). High-quality production of graphene by liquid-phase exfoliation of expanded graphite. Mater. Chem. Phys..

[15] Hou B, Sun H, Peng T, Zhang X, Ren Y (2020). Rapid preparation of expanded graphite at low temperature. New Carbon Mater..

[16] Li M (2020). Highly oriented graphite aerogel fabricated by confined liquid-phase expansion for anisotropically thermally conductive epoxy composites. ACS Appl. Mater. Interfaces.

[17] Zu C, Li L, Qie L, Manthiram A (2015). Expandable-graphite-derived graphene for next-generation battery chemistries. J. Power Sour..

[18] Asif Z, Chen Z, An C, Dong J (2022). Environmental impacts and challenges associated with oil spills on shorelines. JMSE.

[19] Abdellaoui B, Ech-cheikh H, Sadik M, Rachid A, Lissane Elhaq S, Mounadel A (2023). A review on ship-generated oily waste management at ports: current practices, challenges and future directions. Environ. Dev. Sustain..

[20] Al-Majed AA, Adebayo AR, Hossain ME (2012). A sustainable approach to controlling oil spills. J. Environ. Manag..

[21] Yao T (2016). The effect of environmental factors on the adsorption of lubricating oil onto expanded graphite. J. Mol. Liquids.

[22] Singh H, Bhardwaj N, Arya SK, Khatri M (2020). Environmental impacts of oil spills and their remediation by magnetic nanomaterials. Environ. Nanotechnol. Monit. Manag..

[23] Portella CMMA, Tristão MLB, Felcman J (2006). Evaluation of the possibility of contamination of sea water by metal ions present in fuel oil. Fuel.

[24] Ramirez-Corredores, M. M. Metal compounds. In *The Science and Technology of Unconventional Oils*, pp. 223–294 (Elsevier, 2017). 10.1016/B978-0-12-801225-3.00003-6.

[25] Khan H (2013). Metabolic changes of glutathione in human T and B lymphocytes induced by organo-aluminum complex. Afr. J. Pharm. Pharmacol..

[26] Wu K-H, Huang W-C, Hung W-C, Tsai C-W (2021). Sorption and regeneration of expanded graphite/Fe3O4 composite for removal of oil pollution from the water. Mater. Exp..

[27] Kadoshnikov VM (2023). A composite magnetosensitive sorbent based on the expanded graphite for the clean-up of oil spills. Synth. Struct. Prop..

[28] Dimakis N, Flor FA, Salgado A, Adjibi K, Vargas S, Saenz J (2017). Density functional theory calculations on transition metal atoms adsorbed on graphene monolayers. Appl. Surf. Sci..

[29] Appy D (2014). Transition metals on the (0 0 0 1) surface of graphite: Fundamental aspects of adsorption, diffusion, and morphology. Prog. Surf. Sci..

[30] Xu C, Jiao C, Yao R, Lin A, Jiao W (2018). Adsorption and regeneration of expanded graphite modified by CTAB-KBr/H3PO4 for marine oil pollution. Environ. Pollut..

[31] Kaushal, A., Dhawan, S. K., & Singh, V. Determination of crystallite size, number of graphene layers and defect density of graphene oxide (GO) and reduced graphene oxide (RGO). In *Presented at the DAE solid state physics symposium *2018, Hisar, Haryana, India, p. 030106. 10.1063/1.5112945 (2019).

[32] Hristea G, Budrugeac P (2008). Characterization of exfoliated graphite for heavy oil sorption. J. Therm. Anal. Calorim..

[33] Coetzee, D., Militký, J., Wiener, J., & Venkataraman, M. Comparison of the synthesis, properties, and applications of graphite, graphene, and expanded graphite. In Militký, J., & Venkataraman, M., Eds. *Advanced Multifunctional Materials from Fibrous Structures*, vol. 201, Advanced Structured Materials, vol. 201, pp. 71–87 (Springer Nature Singapore, Singapore, 2023). 10.1007/978-981-99-6002-6_4.

[34] Do QC, Choi S, Kim H, Kang S (2019). Adsorption of lead and nickel on to expanded graphite decorated with manganese oxide nanoparticles. Appl. Sci..

